# First record of the Asian bush mosquito, *Aedes japonicus japonicus*, in Italy: invasion from an established Austrian population

**DOI:** 10.1186/s13071-016-1566-6

**Published:** 2016-05-16

**Authors:** Bernhard Seidel, Fabrizio Montarsi, Hartwig P. Huemer, Alexander Indra, Gioia Capelli, Franz Allerberger, Norbert Nowotny

**Affiliations:** Technical Office of Ecology and Landscape Assessment, Nibelungenstrasse 51, 3680 Persenbeug, Austria; Department of Theoretical Biology, University of Vienna, Althanstrasse 14, 1090 Vienna, Austria; Laboratory of Parasitology, Istituto Zooprofilattico Sperimentale delle Venezie, viale dell’Università 10, 35020 Legnaro, Padua Italy; Department of Hygiene, Microbiology & Social Medicine, Medical University of Innsbruck, Fritz-Pregl-Straße 3, 6020 Innsbruck, Austria; Institute for Medical Microbiology and Hygiene, Austrian Agency for Health and Food Safety (AGES), Waehringerstrasse 25a, 1096 Vienna, Austria; Viral Zoonoses, Emerging and Vector-Borne Infections Group, Institute of Virology, University of Veterinary Medicine, Veterinaerplatz 1, 1210 Vienna, Austria; Department of Basic Medical Sciences, College of Medicine, Mohammed Bin Rashid University of Medicine and Health Sciences, Dubai Healthcare City, P.O. Box 505055, Dubai, United Arab Emirates

**Keywords:** *Aedes japonicus*, Asian bush mosquito, Invasive mosquito species, Active spread, First record, Italy, Co-occurrence, *Aedes albopictus*, Asian tiger mosquito

## Abstract

**Background:**

In 2011 we identified the Asian bush mosquito, *Aedes japonicus japonicus* (Theobald, 1901) (Diptera: Culicidae) for the first time in northern Slovenia and in the bordering Austrian federal state of Styria. Between May and July 2012 the distribution area of *Ae. j. japonicus* was already found to be extended westwards into Carinthia and eastwards towards Burgenland and bordering Hungary. In August 2012 the species was first detected in a western province of Hungary. In subsequent years, follow-up field studies demonstrated an active spread westwards throughout Carinthia, reaching the border to northern Italy.

**Findings:**

In July 2015 several aquatic-stage specimens of the species were discovered at three different sites in the Friuli Venezia Giulia region, north-eastern Italy. In September 2015, co-occurrence of *Ae. j. japonicus* and *Aedes albopictus* (Skuse, 1895) was observed in the same sample in that region.

**Conclusions:**

*Ae. j. japonicus* actively extended its geographic range from an established population in Carinthia (Austria) southwards to northern Italy by crossing Alpine ranges. Since *Ae. albopictus* and *Aedes koreicus* (Edwards, 1917) are already well established in northern Italy, it will be pivotal to monitor the consequences of a third invasive mosquito species trying to populate the same geographic region.

## Background

During field surveys in 2011 *Aedes* (*Hulecoeteomyia*) *japonicus japonicus* (Theobald, 1901) (syn. *Hulecoeteomyia japonica*) (Diptera: Culicidae) was found in northern Slovenia and southern Austria [1, 2]. The occurrence of the species had already been reported from other European countries [3–6]. In October 2011 further investigations were conducted in the southeastern districts of Carinthia, where the species was identified at one site outside the city of Lavamünd (46.635667N, 14.952326E, 358 m.a.s.l.). This site is geographically isolated by the surrounding Koralm and Karawanken mountain ranges, with the exception of the River Drau that passes through and connects the central parts of Carinthia with northern Slovenia. Despite intensive mosquito sampling in the Carinthian area, no further *Ae. j. japonicus* individuals were detected there, thus this location can be considered the western limit of this species in Austria until 2011.

During 2012 our investigations demonstated an expansion of the species’ distribution range of at least 25 km from the above-mentioned border location towards central Carinthia. These findings, observed despite relatively harsh weather conditions, e.g. six weeks of constantly frozen ground, during January and February 2012, followed by a rather dry spring (http://www.zamg.ac.at/cms/de/klima/klima-aktuell/klimaspiegel/jahr/klagenfurt_flugh/?jahr=2012), prompted us to hypothesize that the species may occupy entire Carinthia, excluding the mountainous regions, within only a few seasons. Consequently, we also hypothesized that the species will reach the border region to Italy, about 125 km distance, just by expanding to the regions upwards of the River Drau, season by season. In order to test our hypothesis, mosquito sampling was carried out in 2013 and 2014 along the River Drau westwards, where we found the species in the villages Müllern, Gallizien (46.544667N, 14.529391E, 483 m.a.s.l.), Rottenstein (46.560533N, 14.474172E, 417 m.a.s.l.), and Ferlach/Unterloibl (46.508626N, 14.289950E, 507 m.a.s.l.), the most western location towards Italy sampled in 2013/2014. At the latter location, *Ae. j. japonicus* was detected on 10 October 2014.

On 28 July 2015 we found the Asian bush mosquito in Feistritz, Austria (46.563116N, 13.879180E, 488 m.a.s.l.), 13 km west of the 2014 finding in Unterloibl, and in Finkenstein (46.563914N, 13,878716E, 488 m.a.s.l), a village 25 km further west on the way to the Italian border.

## Findings

### First record of *Ae. j. japonicus* in Italy

On 30 July 2015 we identified *Ae. j. japonicus* for the first time in Italy, in three villages along the River Fella: Ugovizza (46.510494N, 13.470631E, 762 m.a.s.l.), Santa Caterina (46.503130N, 13.4000027E, 689 m.a.s.l.) and Pontebba (46.504715N, 13.303009E, 561 m.a.s.l.). The distance between the closest *Ae. j. japonicu*s finding in Austria (Finkenstein) and Italy (Ugovizza) is 36 km.

**Fig. 1:**
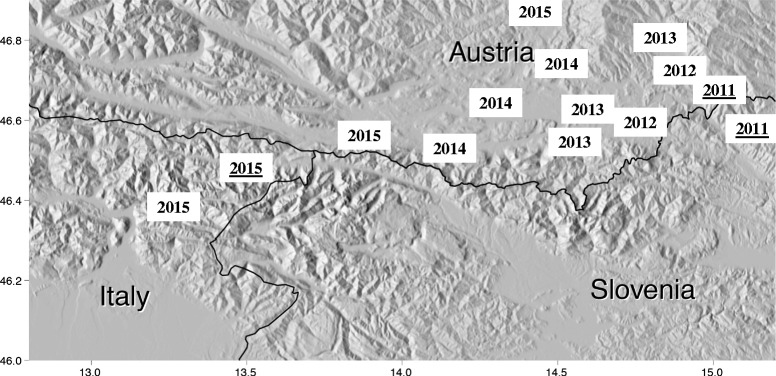
Temporal and spatial spreading of *Aedes japonicus japonicus* from its introduction site from Slovenia to Austria in 2011 to northern Italy in 2015

In Santa Caterina, one larva of *Ae. j. japonicus* was collected. In the same breeding site larvae belonging to *Culex pipiens* (L., 1758), *Cx. hortensis* (Ficalbi, 1889) and *Aedes* (*Dahliana*) *geniculatus* (Olivier, 1791) (syn. *Dahliana geniculata*) were also present. In Ugovizza, samples were taken from several waterfilled plastic buckets and from two used car tires; one *Ae. j. japonicus* pupa was found there, which transformed to an adult female during the road trip. These breeding sites were also monitored in 2013, 2014 and in late April 2015 (three months before its first detection) and were negative for *Ae. j. japonicus* larvae at that time. In Pontebba, eight larvae and eight pupae of *Ae. j. japonicus*, which later completed the metamorphosis into adults, were collected from a small metal water container. Later in the season, in September 2015, *Ae. j. japonicus* was also found in the villages of Resiutta (46.394368N, 13.224345E, 328 m.a.s.l.) and Villanova (46.394368N, 13.224345E, 451 m.a.s.l.). The temporal and spatial active move of *Ae. j. japonicus* from its introduction to Austria in 2011 to northern Italy in 2015 is shown in Fig. 1.

### Genetic confirmation

Three larvae from Pontebba were used for molecular species confirmation. PCR investigations (target gene ND4, primers N4J 8502D-N4N 8944D) [7], revealed amplification products of the expected size (475 bp), and subsequent sequencing [GenBank: KU665643-KU665645] confirmed the species *Ae. j. japonicus,* with 99 % nucleotide (nt) identity (query cover 100 %) to *Ae. j. japonicus* from the USA (GenBank: AF305879), and also 99 % nt identity (query cover 95 %) to *Ae. j. japonicus* from Switzerland, Germany, and The Netherlands (GenBank: KJ958405, KM610233 and KM610232, respectively).

### Co-occurrence of *Ae. j. japonicus* and *Ae. albopictus*

The Asian tiger mosquito *Aedes* (*Stegomyia*) *albopictus* (Skuse, 1895) (syn. *Stegomyia albopicta*) was detected for the first time in Austria in May 2012 in the most southern district of south-eastern Burgenland, bordering Hungary and Slovenia [1]. In September 2012, *Ae. albopictus* larvae were also identified in the village of Angath in a north-eastern district of Tyrol, located in the River Inn valley, only 10 km south of the Bavarian (German) border, but more than 400 km from the first detection site in Burgenland [8]. Despite numerous attempts, *Ae. albopictus* has not been found in Austria after 2012, neither at the 2012 detection sites nor elsewhere. In sharp contrast, *Ae. albopictus* is well established and abundant in Italy, including northern regions [9]. Accordingly, during our monitoring *Ae. albopictus* was also found in several locations of northern Italy, confirming its wide distribution. Interestingly, on 18 September 2015, one larval specimen of *Ae. j. japonicus* was found together with 12 *Ae. albopictus*, three *Cx. pipiens* and one *Cx. hortensis* in the same sample, taken from water-filled used car tires, in the village of Resiutta, 20 km away from the nearest *Ae. j. japonicus* detection site (Pontebba village). This demonstrates for the first time in Italy the co-occurrence of *Ae. j. japonicus* and *Ae. albopictus* at the same breeding site. Both species were also found nearby, six kilometres east of Resiutta upstream of the River Fella in Villanova, which shows an ongoing overlapping of the two species. In northern Italy, a third invasive mosquito species, *Aedes* (*Hulecoeteomyia*) *koreicus* (Edwards, 1917) (syn. *Hulecoeteomyia koreica*), which was discovered in Italy in 2011 [10], is currently expanding its distribution range and is now also present in Friuli Venezia Giulia [11]. Due to the ecological features of this north Italian region, *Ae. j. japonicus* has a good chance to establish itself there.

## Conclusions

*Ae. j. japonicus* extended its geographic range from an established population in Carinthia (Austria) southwards to northern Italy by crossing Alpine ranges. It will be highly interesting to observe how three invasive mosquito species with different ecological needs – *Ae. j. japonicus*, *Ae. albopictus* and *Ae. koreicus* – co-exist in northern Italy in the future. From a Public Health perspective, the introduction of a new mosquito species competent of transmitting pathogens to animals and humans [2] may be an additional challenge for the health system.

## Abbreviations

m.a.s.l.: metres above sea level
nt: nucleotide
